# The *ASIP* Cys‐98 Allele Is Not Associated With Black Coat Color in Water Buffaloes

**DOI:** 10.1002/age.70100

**Published:** 2026-04-10

**Authors:** Júlia Villane Santos de Moraes, Silel Vinicius Simões Andrade Maciel, Ingrid Pereira Pinto Oliveira, Beatriz Bastos Senes, Jackeline Santos Alves, Raphael Bermal Costa, Gregório Miguel Ferreira de Camargo

**Affiliations:** ^1^ Escola de Medicina Veterinária e Zootecnia Universidade Federal da Bahia Salvador Brazil

Polymorphisms in the *ASIP* gene are known to cause variations in coat color in domestic ruminants (Liang et al. 2020; Kumari et al. 2023; Trigo et al. 2021; Dimov et al. 2025; Guo et al. 2022; Fontanesi et al. 2010), as well as in various other livestock and wild species. In cattle, it has been reported that these polymorphisms lead to a darkening of the coat color (Trigo et al. 2021); in buffaloes, a transposition results in a white phenotype (Liang et al. 2020), while a non‐synonymous SNP in exon 3 (XM_025263623.3:c.292C>T) may be associated with black coat color (Kumari et al. 2023). Although the interactions between *MC1R* and *ASIP* have been extensively studied across species, it is noteworthy that the *MC1R* gene exhibits no polymorphism among buffaloes with different coat colors (Cruz et al. 2020). This absence of polymorphism suggests a lack of epistasis with *ASIP*, indicating a distinct genetic architecture in this species. Therefore, the aim of the present study was to perform fine mapping of exon 3 of the *ASIP* gene, which has previously been suggested as a candidate influencing black coat color in buffaloes, in order to assess its association with coat color variation in the Murrah breed.

A total of 57 Murrah buffaloes were used in this study, including four coat colors: brown (*n* = 28), black (*n* = 24), white (*n* = 3), and smoke (*n* = 2) (Figure 1). Among these, 10 dam–offspring pairs were sequenced, one sharing the same color (brown × brown) and nine of different colors, in addition to thirty‐seven unrelated individuals. DNA was extracted from hair follicles. The Ethics Committee on Animal Use of EMVZ‐UFBA approved the project (81/2018). Animals were genotyped using PCR–sequencing, with the primer pair previously described (Kumari et al. 2023). Furthermore, Fisher's exact test was performed in R software to evaluate whether there was an association between the detected SNPs and coat color.

**FIGURE 1:**
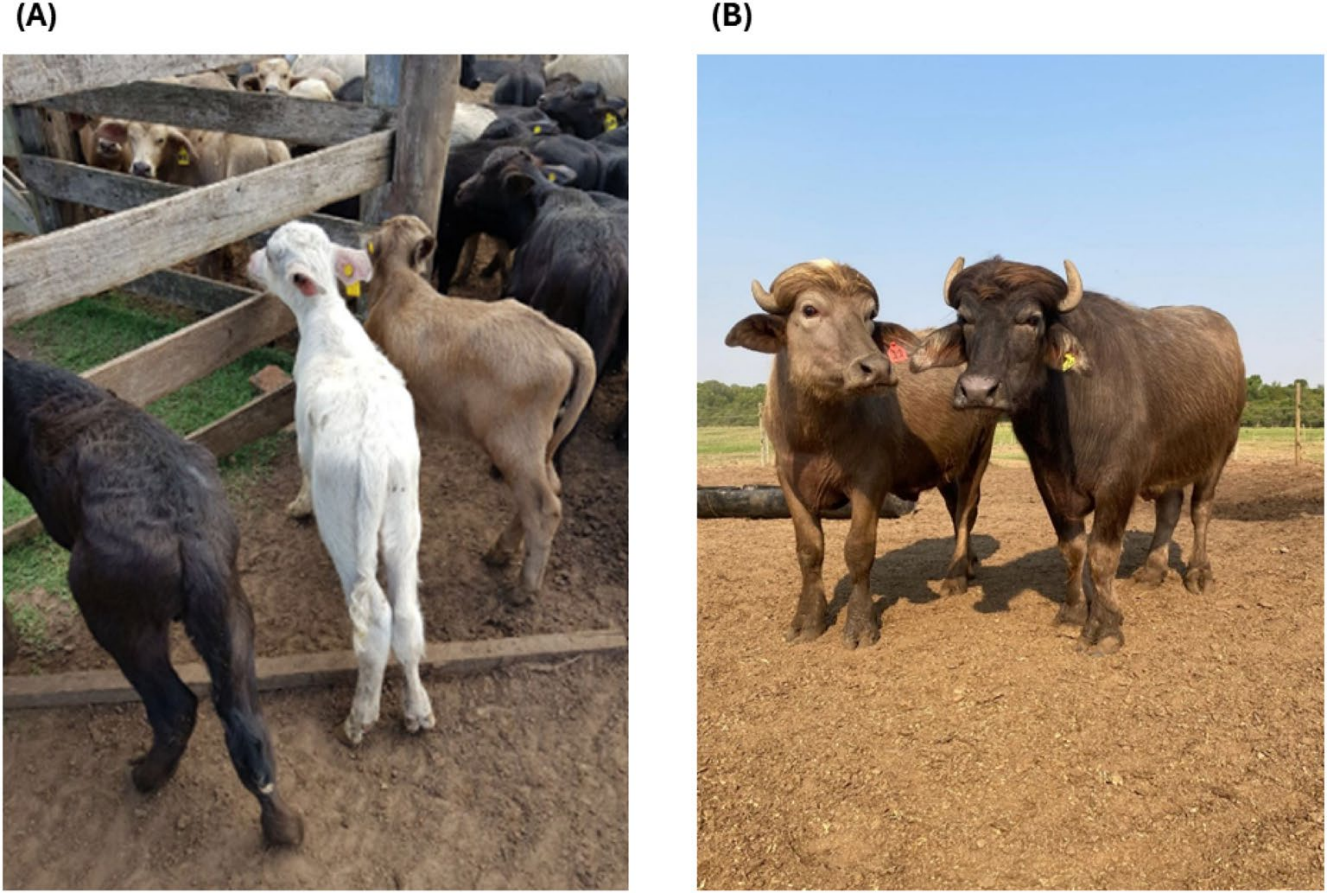
(A) From left to right: Black, white, and brown buffalo calves; (B) On the left: A brown animal; on the right: A smoke animal. (Photo: Davi Matiuzo, Igatu Farm, Itaju‐SP, Brazil).

Sequence analysis of the third coding exon of the *ASIP* gene detected a non‐synonymous SNP at position XM_025263623.3:c.292C>T (XP_025119408.3:p.Arg98Cys) and a synonymous SNP at XM_025263623.3:c.300C>T (XP_025119408.3:p.Ser100). The sequences were deposited in GenBank (accession number: PX549776). Genotypic and allelic frequencies by coat color are presented in Table 1.

**TABLE 1:** Number of animals, genotypic and allelic frequencies of SNPs in the third coding exon of the *ASIP* gene across different coat colors in Murrah buffaloes.

SNP	Genotype	Coat color			
		Brown (*n* = 28)	Black (*n* = 24)	White (*n* = 3)	Smoke (*n* = 2)
XM_025263623.3:c.292C>T	CC	0.46 (*n* = 13)	0.37 (*n* = 9)	0.67 (*n* = 2)	0.5 (*n* = 1)
	CT	0.5 (*n* = 14)	0.46 (*n* = 11)	0.33 (*n* = 1)	0.5 (*n* = 1)
	TT	0.04 (*n* = 1)	0.17 (*n* = 4)	0	0
	**Alleles**				
	C	0.71	0.6	0.83	0.75
	T	0.29	0.4	0.17	0.25
XM_025263623.3:c.300C>T	CC	0.93 (*n* = 26)	1 (*n* = 24)	1 (*n* = 3)	1 (*n* = 2)
	CT	0.07 (*n* = 2)	0	0	0
	TT	0	0	0	0
	**Alleles**				
	C	0.96	1	1	1
	T	0.04	0	0	0

The distribution of genotypic frequencies of the detected SNPs among coat color classes and the absence of association (*p* = 0.54) indicate no evidence of influence of these SNPs on coat color determination. Kumari et al. (2023), Fontanesi et al. (2010) suggested that the high frequency of the TT genotype at c.292 in the Nili‐Ravi and Murrah breeds—both characterized by an intense black coat—could indicate a potential effect of this SNP, given that the frequency of this genotype is lower in lighter‐colored breeds. Although the coat color phenotypes in the present study differ from those reported by Kumari et al., it was observed that the TT genotype was not predominant in black‐coated animals (0.17; Table 1). The genotypic distribution across coat color classes supports the conclusion that this variant is not the causal mutation underlying phenotypic variation. By phenotypic observation, it seems that the three coat colors (black, brown and white) are driven by absence of dominance single gene, to be further investigated.

In conclusion, the polymorphisms identified in the third coding exon of the *ASIP* gene do not appear to affect coat color in Murrah buffaloes. Further studies are warranted to identify the genetic sources responsible for coat color differentiation within this breed.

## Author Contributions

**Júlia Villane Santos de Moraes:** investigation, methodology, formal analysis, writing – review and editing, writing – original draft. **Silel Vinicius Simões Andrade Maciel:** formal analysis, investigation, methodology. **Ingrid Pereira Pinto Oliveira:** investigation, formal analysis, methodology. **Beatriz Bastos Senes:** investigation, methodology, formal analysis. **Jackeline Santos Alves:** investigation, methodology, formal analysis. **Raphael Bermal Costa:** funding acquisition. **Gregório Miguel Ferreira de Camargo:** conceptualization, supervision, writing – review and editing, formal analysis.

## Conflicts of Interest

The authors declare no conflicts of interest.

## Data Availability

The data that support the findings of this study are openly available in Genbank at https://www.ncbi.nlm.nih.gov/nuccore/PX549776, reference number PX549776.

## References

[age70100-bib-0001] Cruz, V. A. R. , J. S. Alves , M. S. Bastos , et al. 2020. “ *MC1R* Gene and Coat Color in Buffaloes.” Animal Genetics 51, no. 2: 345–346. 10.1111/age.12910. 31975429

[age70100-bib-0002] Dimov, D. , M. Kostova , A. Vuchkov , et al. 2025. “Genetic Polymorphisms in Agouti Signaling Protein (ASIP) and Melanocortin 1 Receptor (MC1R) Genes and Their Association With Coat Color in Native Bulgarian Sheep Breeds.” Small Ruminant Research 249: 107517. 10.1016/j.smallrumres.2025.107517.

[age70100-bib-0003] Fontanesi, L. , S. Dall'Olio , F. Beretti , B. Portolano , and V. Russo . 2010. “Coat Colours in the Massese Sheep Breed Are Associated With Mutations in the Agouti Signalling Protein (*ASIP*) and Melanocortin 1 Receptor (*MC1R*) Genes.” Animal 5, no. 1: 8–17. 10.1017/s1751731110001382. 22440696

[age70100-bib-0004] Guo, J. , X. Sun , A. Mao , et al. 2022. “A 13.42‐kb Tandem Duplication at the ASIP Locus Is Strongly Associated With the Depigmentation Phenotype of Non‐Classic Swiss Markings in Goats.” BMC Genomics 23: 437. 10.1186/s12864-022-08672-9. 35698044 PMC9190080

[age70100-bib-0005] Kumari, N. , R. Vasisth , A. Gurao , et al. 2023. “ *ASIP* Gene Polymorphism Associated With Black Coat and Skin Color in Murrah Buffalo.” Environmental and Molecular Mutagenesis 64, no. 5: 309–314. 10.1002/em.22554. 37235680

[age70100-bib-0006] Liang, D. , P. Zhao , J. Si , et al. 2020. “GenomicAnalysis Revealed a Convergent Evolution of LINE‐1 in Coat Color: A Case Study in Water Buffaloes ( *Bubalus bubalis* ).” Molecular Biology and Evolution 38, no. 3: 1122–1136. 10.1093/molbev/msaa279. PMC7947781 33212507

[age70100-bib-0007] Trigo, B. B. , A. T. H. Utsunomiya , A. A. A. D. Fortunato , et al. 2021. “Variants at the *ASIP* Locus Contribute to Coat Color Darkening in Nellore Cattle.” Genetics Selection Evolution 53, no. 1: 40. 10.1186/s12711-021-00633-2. PMC8082809 33910501

